# The diagnostic and prognostic value of LncRNA FAM3D-AS1 expression in nasopharyngeal carcinoma and its effect on tumor progression and cisplatin resistance

**DOI:** 10.1186/s41065-025-00526-0

**Published:** 2025-08-26

**Authors:** Bohan Zhang, Zixiao Chen, Zhinong Wu

**Affiliations:** Department of Clinical Laboratory, Xianning Central Hospital, No. 228, Jingui Road, Xian’an District, Xianning, 437100 China

**Keywords:** Nasopharyngeal carcinoma, LncRNA FAM3D-AS1, Diagnosis, Prognosis, Cisplatin resistance

## Abstract

**Background:**

The location of nasopharyngeal carcinoma (NPC) is relatively hidden. Most patients are diagnosed at the middle or late stage of the disease, having missed the best time for treatment.

**Aim:**

To explore the clinical value of lncRNA FAM3D-AS1 and its effect on tumor progression and cisplatin resistance in nasopharyngeal cancer.

**Materials and methods:**

In this study, a total of 118 NPC patients and 50 healthy volunteers participated. RT-qPCR was analyzed to detect the lncRNA FAM3D-AS1 expression in NPC patients and cells. Receiver Operating Characteristic (ROC curves), Kaplan-Meier (K-M) survival curves and Cox regression analysis were performed to analyze the clinical value value of lncRNA FAM3D-AS1. Cell functional assays were employed to investigate the impact of lncRNA FAM3D-AS1 on biological malignant behavior of NPC and cisplatin resistance.

**Results:**

In NPC patients’ serum and cell lines, the expression of lncRNA FAM3D-AS1 was upregulated. ROC curves with Area Under the Curve (AUC) = 0.920, 95%CI = 0.874–0.965 could be used to distinguish the healthy group from the NPC group. K-M survival curves revealed that NPC patients with high expression of lncRNA FAM3D-AS1 had poor prognosis. Cox regression analysis suggested that lncRNA FAM3D-AS1 was an independent prognostic marker for NPC. Knockdown of lncRNA FAM3D-AS1 expression prohibited NPC cell proliferation, migration, invasion, and cisplatin resistance, but promoted apoptosis.

**Conclusion:**

LncRNA FAM3D-AS1 can be used as a biomarker to diagnose and prognosticate NPC. LncRNA FAM3D-AS1 affects tumor progression and cisplatin resistance in NPC.

## Introduction

Nasopharyngeal Carcinoma (NPC) Nasopharyngeal Carcinoma (NPC) is an epithelial malignant tumor with significant geographic distribution, and about 80% of cases worldwide are concentrated in southern China and Southeast Asia [1, 2]. Epidemiologic studies have demonstrated that its development is closely linked to EBV infection, genetic susceptibility and environmental factors [3, 4]. NPC, the patient’s 5-year survival rate can reach 80%-90% after standardized treatment [5]. Due to the pathological characteristics of this disease, radiotherapy and chemotherapy play a dominant role in the treatment strategy for NPC. Cisplatin is one of the most effective chemotherapy drugs for treating NPC. However, patients who initially responded to cisplatin still develop some degree of resistance later on. However, due to the hidden location of NPC and the lack of specific symptoms in the early stage of NPC, unfortunately, more than 70% of patients are diagnosed at a middle-to-late stage, thus missing the best time for treatment [6]. Imaging tests such as MRI/PET-CT and quantitative EBV DNA tests are routine diagnostic tools in clinical practice, but close to 30% of early-stage patients are missed due to a lack of typical symptoms [7]. Although the survival rate of NPC patients has improved with radiation therapy-based combination therapy, the overall prognosis of NPC patients is poor due to the lack of effective prognostic testing indicators [8, 9]. Existing TNM staging systems have limited value in guiding individualized treatment, especially for cisplatin-resistant patients, with less than 50% accuracy in prognostic prediction [10]. Therefore, there is an urgent need to develop more reliable and valid molecular markers against NPC.

Recently, research into long non-coding RNA (lncRNA) has surged in tumor molecular diagnosis due to its tissue-specific expression, high stability and functional diversity [11]. Compared with protein-based markers, lncRNAs have the following advantages: (a) they can be stably present in body fluids (e.g., plasma, saliva) [12]; (b) The degree of tumor malignancy is closely linked to the expression level [13]; and (c) they can be linked to tumor development via multiple mechanisms, such as the ceRNA network and epigenetic regulation [14, 15]. Recently, a variety of lncRNAs have been shown to be linked to the occurrence and development of NPC. Down-regulation of LncRNA DYNLRB2-AS1 expression inhibits NPC resistance to gemcitabine, and cell proliferation, but promotes apoptosis [16]. LncRNA SUCLG2-AS1 promotes migration and invasion of nasopharyngeal carcinoma cells and inhibits apoptosis and radio-sensitization by regulating SOX2 expression [17]. High lncRNA HOTAIRM1 expression leads to G2/M cell cycle block and cellular DNA damage, increased cell proliferation and decreased apoptosis in nasopharyngeal carcinoma cells after radiotherapy [18]. However, the mechanism of FAM3D-AS1 as an oncogenic lncRNA in NPC has not been systematically elucidated. To delve deeper into the regulatory impact of FAM3D-AS1 on the proliferation and apoptosis of NPC cells, migration and invasion, as well as the specific molecular pathways by which it mediates cisplatin resistance, will provide an important theoretical basis for the novel development of diagnostic markers and strategies for the reversal of drug resistance.

This study mainly studied the significance and value of lncRNA FAM3D-AS1 in the clinical diagnosis and prognosis of NPC. In vitro, experiments elucidated its effects on NPC cell viability, apoptosis, migration and invasion and cisplatin resistance.

## Material and method

### Patients and serum samples

A total of 118 patients who came to Xianning Central Hospital between May 2018 and May 2019 and were diagnosed with positive nasopharyngeal carcinoma were recruited in this study. Fifty healthy volunteers were also recruited. All participants were asked to provide 5 ml of fasting venous blood on the following day of enrolment for the subsequent experiment. Ethical approval for the study was received from the Ethics Committee of Xianning Central Hospital, and written informed consent was obtained from all patients before they participated.

Inclusion criteria: (a) The patients were receiving their first treatment for nasopharyngeal carcinoma. (b) The patient had no other malignancies. (c) The pathological type of the patients was differentiated as nonkeratinizing carcinoma or undifferentiated nonkeratinizing carcinoma. d The patients underwent EVB serum tests. e. Informed consent was obtained from patients and their families. Exclusion criteria: (a) The patients with other malignant tumors or combined with other dual-source cancers were found by admission examination. (b) The patient had other medical conditions that affected the results of this study. (c) The clinicopathological data were incomplete. The patients were followed up for 60 months through outpatient examination, telephone interviews and online follow-up.

### Follow-up

Patients were tracked over a period of 60 months through various means, including outpatient visits, phone interviews, and online follow-ups. Survival time was defined as the period from the start of treatment until death. If the patient was still alive at the last follow-up, the time was calculated up to that point.

### Cell culture

Immortalized nasopharyngeal epithelial cells in humans, NP69 (SUNNCELL), were cultured in TCH-C440 medium. SUNE-1 (BFB), CNE-1 (ATCC), and C666-1 (NTCC) were cultured in DMEM medium. CNE-2 (ATCC) was cultured in RPMI-1640 medium. All media were supplemented with 10% FBS and 1% P/S. In addition, in order to maintain the resistance of CNE-2 and C666-1 cells to cisplatin, in the culture medium of these two cells, 1 µg/mL cisplatin was added. The culture conditions for the cells are 37℃, 5% CO_2_, 95% air and 80% humidity. The cell generations used in the experiment were controlled within the range of P5 to P15.

### Cell transfection

CNE-2 and C666-1 cells were divided into blank group (cells did not do any treatment), si-NC group (transfected with siRNA-NC plasmid), si-lncRNA group (transfected with FAM3D-AS1-siRNA), pc-NC group (transfected with pcDNA-NC plasmid) and pc-lncRNA group (transfected with FAM3D-AS1-pcDNA). The corresponding siRNA plasmids and pcDNA plasmids were transfected for 48 h using Lipofectamine™ 3000 (ThermoFisher, USA). The final concentration of siRNA during transfection was 100 nM, and the final concentration of pcDNA was 50 nM.

### RT-qPCR

RT-qPCR was used to detect the mRNA expression level of lncRNA FAM3D-AS1 in serum and cell lines. Total RNA in serum and cells was extracted using TRIzol reagent, and then the concentration and purity of RNA were determined using a spectrophotometer. Subsequently, the total RNA obtained was reverse transcribed into cDNA using a reverse transcription kit. GAPDH serves as the reference gene. Next, RT-qPCR was performed. Finally, the relative mRNA expression was measured by the 2^−ΔΔCt^ method. The primer sequences are as follows: lncRNA FAM3D-AS1, Forward primer 5’ – GACTGTGGCATCTTTCCAGC − 3’, Reverse primer 3’ - CCTCCTCGGAAACAGAACCC − 5’. GAPDH, Forward primer 5’ - CTCCAGTACCTACCTTACAGGGATT − 3’, Reverse primer 5’ - GCTGCTGGCACCTCCA − 3’.

### MTT assay

Cells digested with trypsin were diluted to 2 × 10^4^ CFU/mL. In 96-well plates, cells were seeded. In each well, 20µL of MTT reagent was added. Next, incubation was continued for 4 hours. Finally, the absorbance value was measured at 490 nm.

### Flow cytometry assay

In the dark, the Annexin V-fluorescein isothiocyanate (5 µL) and PI (10 µL) were used to treat the transfected cells for 10 min. The 1×Binding Buffer was then replenished to 500 µL to maintain the staining environment. The PBS buffer was used to wash the cells, the level of apoptosis was detected by CytoFLEX.

### Transwell assay

The first was the migration experiment, in the transwell upper chamber, 5 × 10^5^ CNE-2 and C666-1 cells cultured in serum starvation were seeded. To assess cell invasion, culture plates were precooled on ice and then precoated with Matrigel on top of the transwell upper chamber. During both experiments, a culture medium containing 10% serum was added to the lower chamber of the transwell. After 24 h, 4% paraformaldehyde was used to fix cells for 20 min and 0.1% crystal violet was used to stain cells for 15 min. Finally, five independent visual fields were selected for statistical analysis.

### Cisplatin resistance assay

1 × 10⁴ CNE-2 and C666-1 cells per well were treated with 0, 3, 6, 9, and 12 µg/mL cisplatin for 24 h, respectively. Cell viability was then measured by MTT assay as described above.

### Combination therapy assay

CNE-2 and C666-1 cells were divided into a control group (cells did not do any treatment), cisplatin group (6 µg/mL cisplatin was used to treat cells), si-lncRNA group (FAM3D-AS1-siRNA was used to transfect cells) and cisplatin + si-lncRNA group (cells transfected with FAM3D-AS1-siRNA and treated with 6 µg/mL cisplatin). Then, the cell viability of each group was measured as described above by the MTT assay.

### Statistical analysis

All experimental data were shown as mean ± SD. SPSS 27.0.1 and GraphPad Prism 10.1.2 were used for data analysis. The inter-group comparisons were conducted using the student’s t-test, one-way analysis of variance, or two-way analysis of variance. The chi-square test was employed to explore the association between clinicopathological characteristics and FAM3D-AS1 expression. The diagnostic value of FAM3D-AS1 was assessed using the ROC curve, while its prognostic significance was evaluated through Kaplan-Meier analysis and Cox regression analysis. All the experiments were conducted more than three times, and *p* < 0.05 indicated statistical significance.

## Results

### LncRNA FAM3D-AS1 is up-regulated in the serum of NPC patients and cell lines

**Fig. 1:**
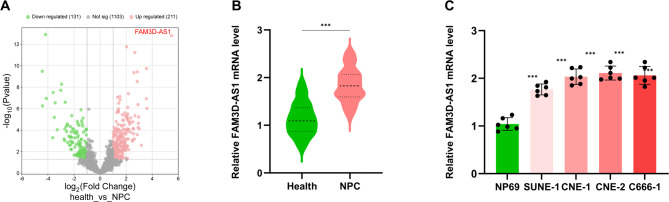
LncRNA FAM3D-AS1 is up-regulated in the serum of NPC patients and cell lines. (**A**) Volcano plot of GSE227541in healthy compared to nasopharyngeal carcinoma. FAM3D-AS1 has the highest llog2Fold Change value. (**B**) LncRNA FAM3D-AS1 expression was upregulated in nasopharyngeal carcinoma patients compared to healthy participants. (**C**) LncRNA FAM3D-AS1 expression was upregulated in nasopharyngeal carcinoma cells (SUNE-A, CNE-1, CNE-2 and C666-1) compared to healthy human nasopharyngeal carcinoma epithelial cells (NP69). ***p < 0.001

The volcano plot illustrating differentially expressed genes in the GSE227541 dataset was generated using the Weishengxin online platform (https://www.bioinformatics.com.cn/). The volcano plot revealed that 131 genes were down-regulated and 211 were up-regulated in the healthy group compared to the NPC group. (Fig. 1A) Among all the analyzed lncRNAs, FAM3D-AS1 exhibited the highest log2FoldChange value; therefore, it was selected as the primary research focus (Fig. 1A). RT-qPCR was employed to quantify the lncRNA FAM3D-AS1 expression levels in both serum samples and cell lines. The results show that the expression level of lncRNA FAM3D-AS1 was increased in the serum of NPC patients compared with healthy controls (*p* < 0.01, Fig. 1B). Similarly, the expression level of lncRNA FAM3D-AS1 was upregulated in SUNE-1, CNE-1, CNE-2 and C666-1 cells compared with NP69 cells (*p* < 0.01, Fig. 1C).

### The diagnostic and prognostic value of LncRNA FAM3D-AS1

**Fig. 2:**
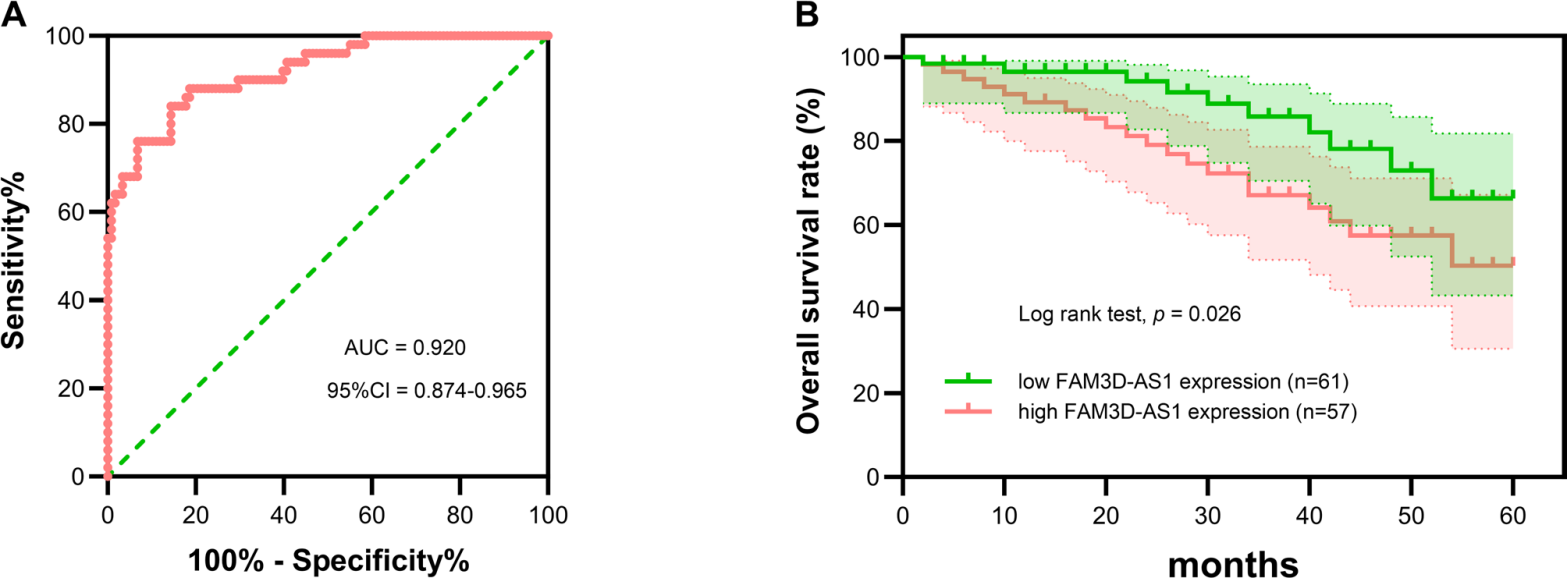
The diagnostic and prognostic value of lncRNA FAM3D-AS1. (**A**) ROC curve was performed to evaluate the diagnostic value of lncRNA FAM3D-AS1. Its AUC was 0.920, with a 95% confidence interval of 0.874–0.965. (**B**) K-M survival curve was used to assess the prognostic value of lncRNA FAM3D-AS1. The low expression of lncRNA FAM3D-AS1 NPC patients had a poor prognosis. ***p < 0.001

ROC curve was performed to evaluate the lncRNA FAM3D-AS1’s clinical diagnostic value. The AUC of lncRNAFAM3D-AS1 was 0.920 (95%CI: 0.874–0.965), suggestting that lncrNAFAM3D-AS1 could better distinguish the healthy group from the NPC group (*p* < 0.01, Fig. 2A). When the optimal cut-off value was selected, the ROC curve’s sensitivity and specificity were 85.6% and 84.0%, respectively (Fig. 2A). The aforementioned findings suggest that lncRNA FAM3D-AS1 holds significant clinical potential in NPC diagnosis. Based on the lncRNA FAM3D-AS1 average expression level of NPC patients, 118 NPC patients were separated into high FAM3D-AS1 expression (*n* = 57) and low FAM3D-AS1 expression (*n* = 61). The chi-square test showed that EVB, TNM Stage and histological grade were significantly correlated with lncRNA FAM3D-AS1 expression. Table 1. In addition, the K-M survival curves showed that there was a significant difference in survival time between high FAM3D-AS1 expression group and low FAM3D-AS1 expression group (*p* < 0.01, Fig. 2B). Additionally, The low expression of lncRNA FAM3D-AS1 NPC patients had a poor prognosis (*p* < 0.01, Fig. 2B). Meanwhile, Cox regression analysis shows that lncRNA FAM3D-AS1 was an predictor of patient survival time (HR = 2.834, 95%CI: 1.091–7.365, *p* = 0.032; Table 2).

**Table 1:** Relationship between clinical characteristics and FAM3D-AS1 in NPC patients

Characteristics	Patients Number	FAM3D-AS1 serum expression		*p*
		High (*n* = 57)	Low (*n* = 61)	
Age				0.864
≥ 45	57	28	29	
<45	61	29	32	
Sex				0.100
Male	57	32	25	
Female	61	25	36	
EBV				**0.017**
Positive	59	35	24	
Negative	59	22	37	
Smoking				0.363
Yes	57	30	27	
No	61	27	34	
Family History				0.142
Yes	58	32	26	
No	60	25	35	
TNM Stage				**0.028**
I+II	62	24	38	
III+VI	56	33	23	
Histological grade				**0.045**
Undifferentiated	55	32	23	
Differentiated	63	25	38	

EBV: Epstein-Barr virus

**Table 2:** Multiple factor Cox regression analysis to evaluate the prognostic value of FAM3D-AS1

Characteristics	HR	95%CI	*p*
FAM3D-AS1	2.834	1.091–7.365	**0.032**
Age	1.229	0.547–2.763	0.618
Sex	1.235	0.525–2.909	0.629
EBV	1.748	0.772–3.958	0.181
Smoking	1.304	0.562–3.026	0.537
Family History	1.229	0.538–2.808	0.625
TNM Stage	1.399	0.596–3.284	0.411
Histological grade	1.259	0.563–2.815	0.576

EBV: Epstein-Barr virus

### Effect of LncRNA FAM3D-AS1 on NPC cell lines

**Fig. 3:**
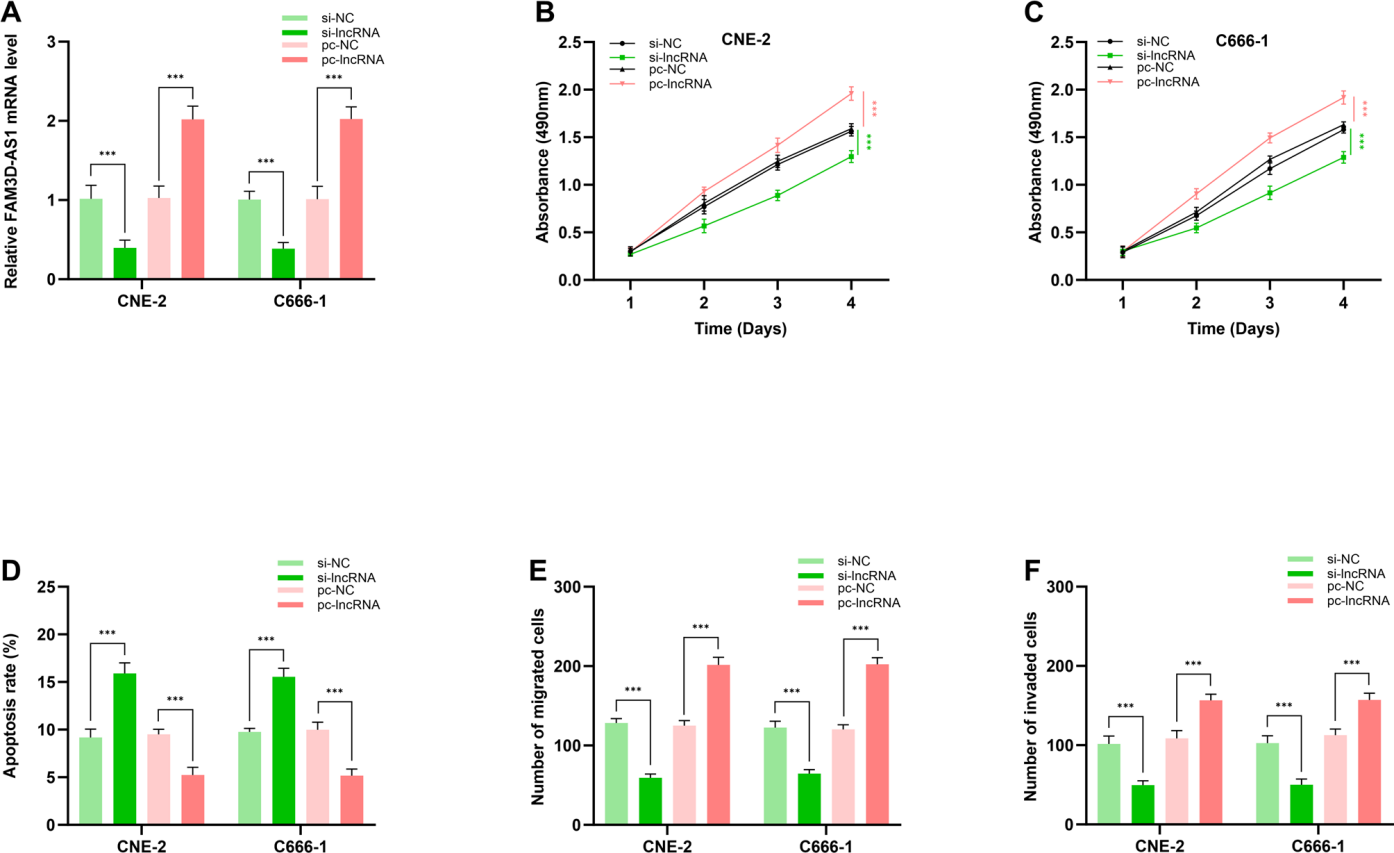
Effect of lncRNA FAM3D-AS1 on NPC cell lines. (**A**) RT-qPCR was used to test the lncRNA FAM3D-AS1 expression. MTT was used to test proliferation of (**B**) CNE-2 and (**C**) C666-1 cells after knockdown or overexpression of lncRNA FAM3D-AS1. (**D**) Flow cytometry was performed to test apoptosis of CNE-2 and C666-1 cells after knockdown or overexpression of lncRNA FAM3D-AS1. Transwell assay was performed to test (**E**) migration and (**F**) invasion of CNE-2 and C666-1 cells after knockdown or overexpression of lncRNA FAM3D-AS1. *** *p* < 0.001

RT-qPCR showed that the lncRNA FAM3D-AS1 expression level was successfully down-regulated and up-regulated after si-lncRNA and pc-lncRNA were transfected (*p* < 0.01, Fig. 3A). After the expression of lncRNA FAM3D-AS1 was down-regulated or up-regulated by RNA transfection, the proliferation activity of CNE-2 and C666-1 cells was detected by MTT assay at 1, 2, 3, and 4 days after transfection. The results show that lncRNA FAM3D-AS1 knockdown attenuated the proliferation ability of CNE-2 and C666-1 cells compared with the negative control group (*p* < 0.01, Fig. 3B-C). Interestingly, up-regulation of lncRNA FAM3D-AS1 enhanced the proliferation ability of both cell lines (*p* < 0.01, Fig. 3B-C). Similarly, the results of apoptosis assay showed that inhibition of lncRNA FAM3D-AS1 increased the apoptosis rate of CNE-2 and C666-1 cells (*p* < 0.01, Fig. 3D). However, up-regulation of lncRNA FAM3D-AS1 expression reduced the apoptosis rate of both cell lines (*p* < 0.01, Fig. 3D). Notably, the results of transwell assay shows that inhibition of lncRNA FAM3D-AS1 expression reduced the number of migration and invasion of CNE-2 and C666-1 cells (*p* < 0.01, Fig. 3E-F). Interestingly, the upregulation of lncRNA FAM3D-AS1 expression increased the number of migrations and invasions of these two cell types (*p* < 0.01, Fig. 3E-F).

### Effect of LncRNA FAM3D-AS1 on cisplatin resistance

**Fig. 4:**
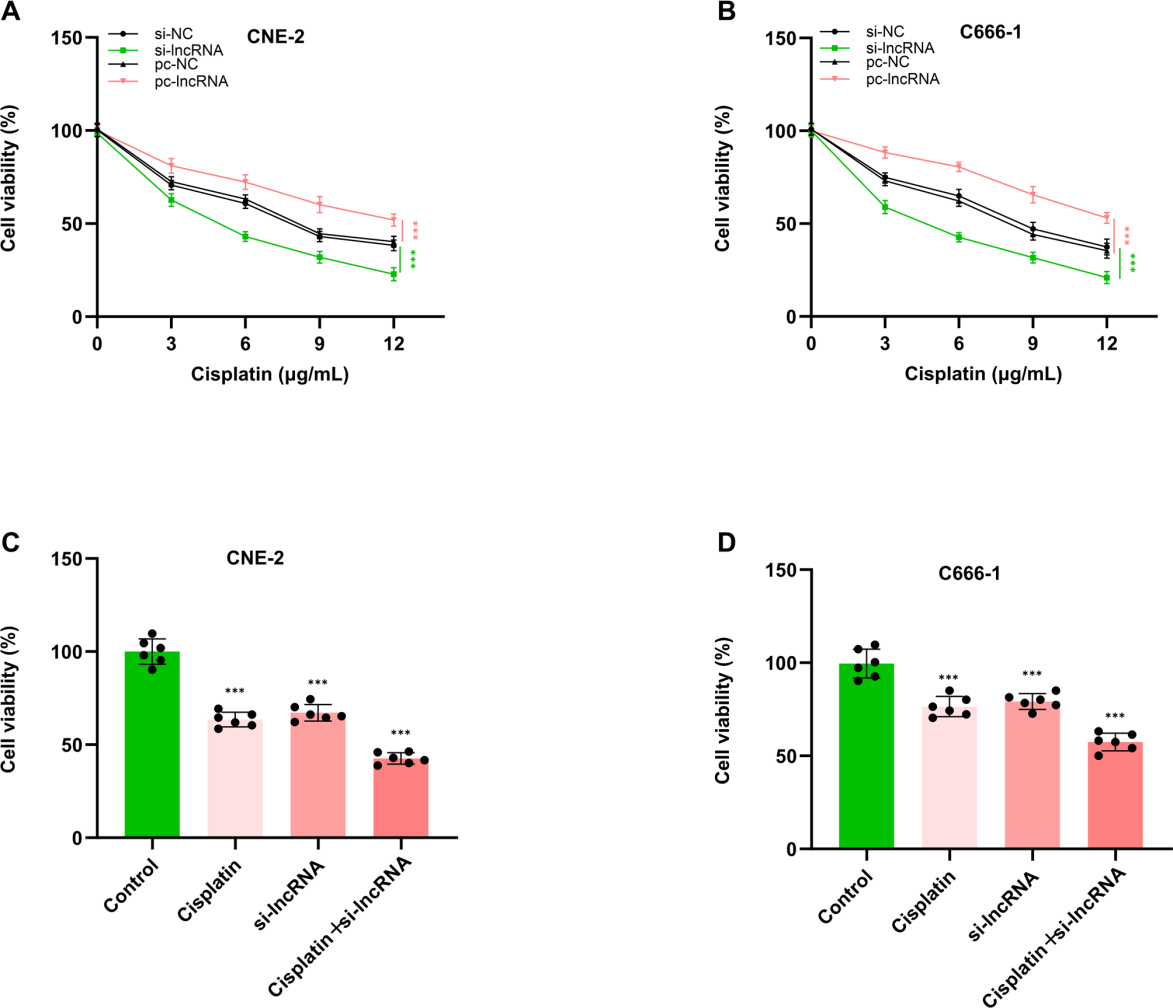
Effect of lncRNA FAM3D-AS1 on cisplatin resistance. Alteration of cisplatin resistance in (**A**) CNE-2 and (**B**) C666-1 cells after knockdown or overexpression of lncRNA FAM3D-AS1. Effects of cisplatin, si-lncRNA and cisplatin combined with si-lncRNA on the viability of (**C**) CNE-2 and (**D**) C666-1 cells. *** *p* < 0.001

To investigate the effect of lncRNA FAM3D-AS1 on the resistance of CNE-2 and C666-1 cells to cisplatin, we examined the cell activity of these two cells after down-regulating and up-regulating lncRNA FAM3D-AS1 expression by RNA transfection. The results show that the inhibition of lncRNA FAM3D-AS1 reduced the resistance of CNE-2 and C666-1 cells to cisplatin (*p* < 0.01, Fig. 4A-B). However, upregulation of lncRNA FAM3D-AS1 expression enhanced cisplatin resistance in both cell lines (*p* < 0.01, Fig. 4A-B). Next, we further demonstrated the effect of cisplatin combined with targeted lncRNA FAM3D-AS1 to alleviate NPC by in vitro experiments. The results show that both cisplatin and down-regulation of lncRNA FAM3D-AS1 expression reduced the cell viability compared with the blank control (*p* < 0.01, Fig. 4C-D). Notably, cisplatin combined with lncRNA FAM3D-AS1 could further reduce the cell viability (*p* < 0.01, Fig. 4C-D).

## Discussion

NPC is a prevalent form of epithelial cancer [19]. Owing to its anatomical location, the complexity of surgical intervention, and the challenges associated with achieving complete tumor resection, it poses significant difficulties for clinical diagnosis and prognosis [20]. Further enhancement of the diagnosis and treatment of NPC has been the focus of recent research. In addition, starting at the molecular level is the basis for innovative NPC treatment strategies. Recent studies have shown that lncRNAs exhibit outstanding value in precise diagnostic and prognostic markers, targeted therapy for multiple diseases, reversal of drug resistance, and immune microenvironment regulation [21]. For example, it has been shown that the lncRNA MALATA1 has been studied in a variety of cancers, where it has been shown to play an important role in cancer diagnosis and prognosis [22]. Another study showed that lncRNA CCDC144NL-AS1 was aberrantly expressed in ovarian cancer, gastric cancer, hepatocellular carcinoma, non-small cell lung cancer and osteosarcoma and that it may be a tumor prognostic marker and an important target for therapy [23]. In this study, we mined the GEO database for the dataset GSE227541. By plotting the volcano plot of this dataset, we observe that lncRNA FAM3D-AS1 was abnormally expressed in NPC patients compared with healthy individuals, and it had the highest log2FoldChange value. Therefore, we speculate that lncRNA FAM3D-AS1 may be involved in the occurrence and progression of NPC. Using RT-qPCR, we observed that the lncRNA FAM3D-AS1 was raised in NPC patients and cells. In recent years, a variety of lncRNAs have been successively proven to be highly potential biomarkers in disease diagnosis and prognosis. LncRNA CAIF has been proven to be downregulated in cardiomyopathy and plays an important diagnostic role in the diagnosis and prognosis of this disease [24]. LncRNA C-497E21.4 in the serum of gastric cancer patients has been demonstrated to be significantly associated with poor prognosis and may be a promising biomarker for the diagnosis and prognostic evaluation of gastric cancer [25]. In this study, the AUC of the lncRNA FAM3D-AS1ROC curve was 0.920, and its sensitivity and specificity were 85.6% and 84%, respectively. The result suggests that the diagnostic model can reliably and sensitively distinguish the healthy group from the NPC group. In addition, survival analysis indicates that high expression of lncRNA FAM3D-AS1 in patients has a poor prognosis. These findings suggest that lncRNA FAM3D-AS1 may be a highly promising biomarker for the clinical diagnosis and prognosis of NPC.

The key role that LncRNAs play in the regulation of cancer cells’ malignant biological behaviour is well-known [26]. Recent studies have shown that lncRNAs affect tumor proliferation, apoptosis, migration, and invasion through a variety of molecular mechanisms [27]. For example, lncRNA CASC9 targeting miR-424-5p affects the proliferation, apoptosis, and migration of hepatocellular carcinoma cells [28]. Knockdown of the lncRNA ELFN1-AS1 inhibits G6PD expression and thus tumor growth in mice transplanted into the body [29]. Furthermore, a study has shown that lncRNA FAM3D-AS1 inhibits the development of colorectal cancer by acting through the NF-kB signaling pathway [30]. However, the effect of lncRNA FAM3D-AS1 on NPC has rarely been reported. In our study, the lncRNA FAM3D-AS1 expression level was successfully knocked down and overexpressed in CNE-2 and C666-1 cells using siRNA and pcDNA. We found that the inhibition of lncRNAFAM3D-AS1 inhibited cell proliferation, migration and invasion, but promoted cell apoptosis. However, overexpression of lncRNA FAM3D-AS1 had the opposite effect on the malignant behavior of these NPC cells. Therefore, we hypothesized that lncRNA FAM3D-AS1 plays an essential role in the genesis and development of NPC.

We all know that most patients with advanced NPC cannot benefit from surgery and have a poor prognosis [31]. Radiotherapy and chemotherapy are crucial in the treatment strategy of NPC [32]. Cisplatin is considered to be one of the most effective chemotherapy drugs for NPC [33]. Studies have shown that patients who are initially sensitive to cisplatin will still develop resistance to cisplatin. Therefore, in this study, we investigated the effect of lncRNA FAM3D-AS1 on cisplatin resistance. We observed that the knockdown of lncRNA FAM3D-AS1 reduced cisplatin resistance. However, overexpression of the lncRNA FAM3D-AS1 produced the opposite effect. In addition, we found that the knockdown of lncRNA FAM3D-AS1 increased the sensitivity of CNE-2 and C666-1 to cisplatin. These findings will provide new ideas for radiotherapy and chemotherapy for NPC patients.

## Conclusion

This study elucidates the value of lncRNA FAM3D-AS1 in the diagnosis and prognosis of NPC and its novel role in cisplatin resistance. LncRNA FAM3D-AS1 is involved in NPC tumorigenesis and progression by regulating NPC tumor proliferation, apoptosis, migration and invasion.

The mechanism exploration in this study was conducted at the cellular model level. The artificial culture conditions cannot truly simulate the dynamic balance within the body, lacking neuro-humoral regulation, intercellular interactions, as well as blood or lymph supply. Furthermore, the metabolism of drugs in the body may be active, but it is difficult to simulate the systemic processes, such as liver metabolism, in vitro. The limitations of the above cell models may lead to an inaccurate assessment of cancer. Therefore, this research result still requires further verification through in vivo or clinical experiments in the future.

## Data Availability

No datasets were generated or analysed during the current study.
